# Neural circuit regulation by identified modulatory projection neurons

**DOI:** 10.3389/fnins.2023.1154769

**Published:** 2023-03-17

**Authors:** Dawn M. Blitz

**Affiliations:** Department of Biology and Center for Neuroscience, Miami University, Oxford, OH, United States

**Keywords:** central pattern generator, neuropeptide, feedback, neuromodulation, neural circuit, modulatory projection neuron

## Abstract

Rhythmic behaviors (e.g., walking, breathing, and chewing) are produced by central pattern generator (CPG) circuits. These circuits are highly dynamic due to a multitude of input they receive from hormones, sensory neurons, and modulatory projection neurons. Such inputs not only turn CPG circuits on and off, but they adjust their synaptic and cellular properties to select behaviorally relevant outputs that last from seconds to hours. Similar to the contributions of fully identified connectomes to establishing general principles of circuit function and flexibility, identified modulatory neurons have enabled key insights into neural circuit modulation. For instance, while bath-applying neuromodulators continues to be an important approach to studying neural circuit modulation, this approach does not always mimic the neural circuit response to neuronal release of the same modulator. There is additional complexity in the actions of neuronally-released modulators due to: (1) the prevalence of co-transmitters, (2) local- and long-distance feedback regulating the timing of (co-)release, and (3) differential regulation of co-transmitter release. Identifying the physiological stimuli (e.g., identified sensory neurons) that activate modulatory projection neurons has demonstrated multiple “modulatory codes” for selecting particular circuit outputs. In some cases, population coding occurs, and in others circuit output is determined by the firing pattern and rate of the modulatory projection neurons. The ability to perform electrophysiological recordings and manipulations of small populations of identified neurons at multiple levels of rhythmic motor systems remains an important approach for determining the cellular and synaptic mechanisms underlying the rapid adaptability of rhythmic neural circuits.

## 1. Introduction

Rhythmic motor behaviors are generated by central nervous system (CNS) circuits called central pattern generators (CPGs) (Bucher et al., 2015). Although CPGs can produce rhythmic output without rhythmic input, modulatory input is often required to configure CPGs into an active state. Additionally, beyond simply turning on or off, CPGs are often “multifunctional,” in that they produce different outputs to adapt to changes in the internal and external environments (Briggman and Kristan, 2008; Benjamin, 2012; Daur et al., 2016; Marder et al., 2022). In some cases, the source of modulation is intrinsic to the CPG and a necessary component of motor output (Katz, 1998). However, many sources originate outside the CPG, including sensory inputs, hormones, and modulatory projection neurons (PNs), i.e., neurons which originate in higher order CNS regions and project to CPGs (Rosen et al., 1991; Briggman and Kristan, 2008; Nusbaum, 2008; Hsu and Bhandawat, 2016).

Small circuits, particularly those underlying rhythmic behaviors, with their identified neurons, have enabled many important insights into circuit function and plasticity (Calabrese et al., 2016; Cropper et al., 2018; Katz and Quinlan, 2019; Marder et al., 2022). Similar to the accessibility of identified circuit neurons, several invertebrate preparations also have relatively small populations of modulatory PNs which are accessible to electrophysiological approaches (Rosen et al., 1991; Heinrich, 2002; Mesce et al., 2008; Nusbaum, 2008). PN populations range from ∼20 pairs in crab and mollusk feeding systems to ∼200–500 pairs targeting the insect ventral nerve cord (Rosen et al., 1991; Coleman et al., 1992; Hsu and Bhandawat, 2016; Namiki et al., 2018). Comparable PN populations in vertebrates are typically larger, include heterogeneous types, and can be distributed across multiple nuclei (Garcia et al., 2011; Sharples et al., 2014; Ruder and Arber, 2019; Flaive et al., 2020). While technological advances are increasing the ability to control vertebrate neuron populations *in vitro* and *in vivo*, cellular-level experimental access to modulatory PNs and a fully described motor circuit connectome remains challenging in many vertebrate preparations (Kim et al., 2017; Leiras et al., 2022). Here, I will focus on lessons learned from several small, invertebrate motor systems, regarding the cellular mechanisms by which modulatory PNs alter CPG output, and how their activity is regulated. Much additional work on descending motor control, including fast activation of escape behaviors, and large-scale genetic approaches investigating insect descending neurons is beyond the scope of this article (Cande et al., 2018; Herberholz, 2022).

## 2. Modulatory projection neurons alter CPG output

### 2.1. Bath-application vs. neuronal-release

**FIGURE 1:**
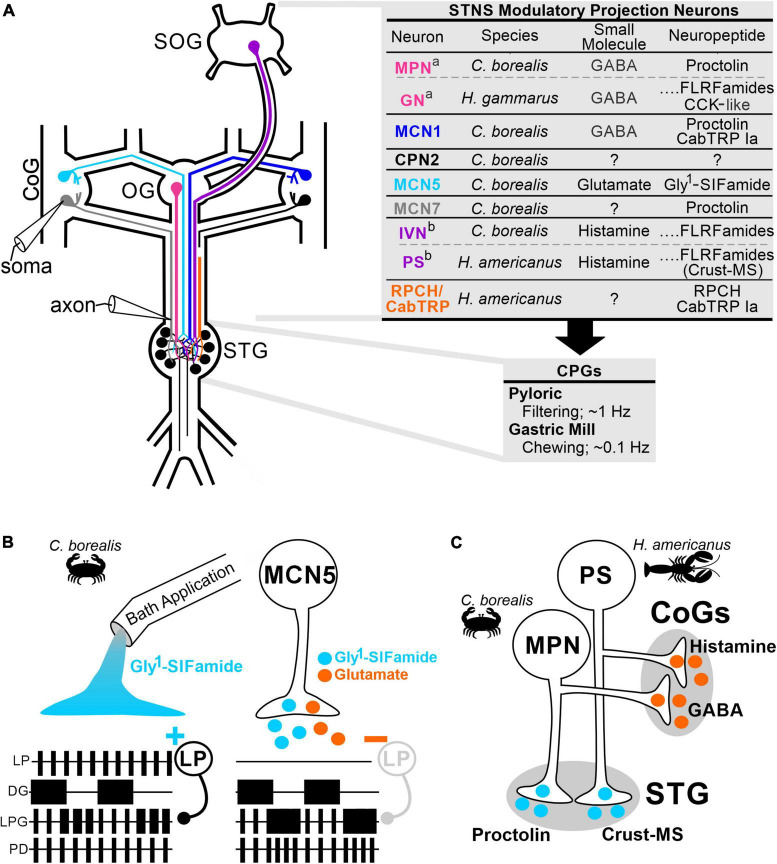
Identified modulatory projection neurons reveal cooperative and divergent actions contributing to distinctions between bath-applied and neuronally-released modulator. **(A)** The crustacean stomatogastric nervous system (STNS) includes the pyloric (food filtering, ∼1 Hz) and gastric mill (food chewing, ∼0.1 Hz) CPGs within the stomatogastric ganglion (STG). Modulatory PNs originating in the oesophageal (OG), the paired commissural ganglia (CoGs), and the supraoesophageal ganglion (SOG) project to and modulate the CPGs. Intracellular recordings of modulatory PNs can be made at the soma in the SOG, CoG, or OG, and axon terminals near the entrance to the STG (electrode symbols). Most modulatory PNs contain small molecule and neuropeptide co-transmitters as listed in the upper table. ^a,b^Some analogous modulatory neurons in different species (lobster, *Homarus gammarus*, *H. americanus*; crab, *Cancer borealis*) contain the same co-transmitters, and others contain different complements. All PNs listed occur as pairs, either as a single copy in each CoG (MCN1/5/7, CPN2), or in the same location (OG: MPN/GN; SOG: IVN/PS), however they are drawn as single neurons for clarity. **(B)** Ionotropic co-transmitter actions are necessary for full expression of metabotropic actions. In *C. borealis*, the modulatory PN MCN5 elicits a motor pattern that includes dual-network activity in the LPG neuron (shorter duration, faster pyloric-timed bursts alternating with slower gastric mill-timed bursts). Pyloric network activity is evident in LP and PD neuron activity, gastric mill network activity is represented by DG neuron activity. Neuron activity is schematized as extracellular recordings with each box representing a burst of action potentials. Bath application of the MCN5 neuropeptide Gly^1^-SIFamide mimics some but not all MCN5 actions. In particular, Gly^1^-SIFamide excites the pyloric LP neuron (+) whereas MCN5 inhibits LP (–). The increased LP activity during Gly^1^-SIFamide application inhibits the LPG neuron, preventing it from fully participating in the slower gastric mill network, note the extended duration LPG bursts alternating with DG that do not fully merge into a gastric mill-timed burst. MCN5 inhibits LP (gray) *via* its co-transmitter glutamate, which is essential for LPG to fully participate in the gastric mill network *via* Gly^1^-SIFamide effects (Blitz et al., 2019; Fahoum and Blitz, 2021). **(C)** Spatially divergent co-transmitter actions occur in modulatory PNs in the STNS. The MPN and PS neurons use their peptide transmitters (proctolin and crust-MS, respectively) on pyloric and gastric mill CPGs in the STG, but their small molecule transmitters (GABA and histamine, respectively) in the CoGs. It is not known whether there is differential trafficking or other explanations for these segregated co-transmitter actions (Nusbaum and Marder, 1989a; Blitz and Nusbaum, 1999; Kwiatkowski et al., 2013). Species used in the referenced studies are indicted in each panel. Neuron/transmitter identification in panel **(A)**: (Nusbaum and Marder, 1989a; Coleman and Nusbaum, 1994; Norris et al., 1994, 1996; Blitz and Nusbaum, 1999; Blitz et al., 1999, 2019; Meyrand et al., 2000; Swensen et al., 2000; Thirumalai and Marder, 2002; Christie et al., 2004; Kwiatkowski et al., 2013; Fahoum and Blitz, 2021).

Early studies primarily using bath-applied neuromodulators, but also stimulation of identified modulatory PNs, demonstrated that there is considerable flexibility in the strength and pattern of neuronal activity, as well as in which CPG(s) the neurons are participating (Hooper and Marder, 1984; Kuhlman et al., 1985; Flamm and Harris-Warrick, 1986; Dickinson et al., 1990; Harris-Warrick and Marder, 1991; Ramirez and Pearson, 1991; Marder, 2012). Although bath-application continues to provide insights into circuit modulation, bath-applied modulator actions range from very similar to neuronally-released modulator, to only mimicking some effects, to having distinct, even opposite effects (Marder, 2012; Nusbaum et al., 2017). The small numbers and exceptional experimental access afforded by invertebrate modulatory neurons have revealed several explanations for distinctions between bath-applied and neuronally-released modulators. The crustacean stomatogastric nervous system (STNS), is particularly useful because the transmitters, intrinsic properties, and synaptic connections are identified for the ∼30 neurons comprising two feeding-related CPGs (pyloric, gastric mill) (Figure 1A; Marder and Bucher, 2007; Daur et al., 2016). Additionally, identified modulatory PNs are amenable to intra-somatic and intra-axonal recordings, and identification of their (co-)transmitter content allows for direct comparison of bath-applied vs. neuronally-released neuromodulators (Figure 1A; Nusbaum and Marder, 1989a; Coleman and Nusbaum, 1994; Stein, 2009; Kwiatkowski et al., 2013; Nusbaum et al., 2017).

### 2.2. Co-transmission

Modulatory CPG inputs, including PNs, use metabotropic receptors and second messenger signaling to alter intrinsic and synaptic properties of circuit neurons to select different outputs (Katz and Calin-Jageman, 2009; Nadim and Bucher, 2014). However, they often also use rapid ionotropic transmission. Co-transmission is ubiquitous and a likely contributor to distinctions between modulatory neuron activation and bath-application. Co-transmitter complements include neuropeptide plus classical and/or amine small molecule transmitters, or multiple small molecule transmitters (Nusbaum et al., 2017; Nässel, 2018; Trudeau and El Mestikawy, 2018; Svensson et al., 2019; Eiden et al., 2022). One or more neuropeptides plus a small molecule transmitter is common in modulatory PNs targeting CPGs (Figure 1A; Schlegel et al., 2016; Nusbaum et al., 2017; Nässel, 2018).

Neuropeptide and small molecule co-neurotransmitter actions range from varying degrees of convergence, to complementary, to entirely divergent (Thirumalai and Marder, 2002; Nusbaum et al., 2017; Nässel, 2018; Florman and Alkema, 2022). In the crab STNS, a modulatory PN (MCN5) switches the CPG neuron LPG from pyloric-only network participation to dual-network (pyloric plus gastric mill) activity *via* its neuropeptide Gly^1^-SIFamide (Figure 1B; Fahoum and Blitz, 2021; Snyder and Blitz, 2022). However, bath applied Gly^1^-SIFamide excites the pyloric CPG neuron LP, which inhibits LPG and prevents it from fully expressing dual-network activity. This Gly^1^-SIFamide excitation of LP is opposite of MCN5 actions (Figure 1B; Fahoum and Blitz, 2021). MCN5-released Gly^1^-SIFamide can elicit the switch in LPG activity due to co-released glutamate inhibiting the LP neuron that would otherwise interfere with LPG switching into dual-network activity (Figure 1B). Thus, ionotropic classical transmitter actions are essential for metabotropic neuropeptide actions to be fully expressed. Conversely, in *Aplysia* feeding, ionotropic actions are enhanced by metabotropic receptor-mediated co-transmitter actions. The feeding motor pattern activated by the modulatory PN CBI-2 changes over time, due to CBI-2 modulation of its cholinergic synaptic transmission onto feeding motor neurons (Koh et al., 2003). The time-dependent effects on the motor pattern and enhanced fast cholinergic synaptic transmission are mimicked by either of the CBI-2 peptide co-transmitters (CP2, FCAP). However, the cooperative peptide effects are distinct, with CP2 and FCAP increasing quantal content versus size, respectively (Koh et al., 2003). Intracellular recordings from identified modulatory PNs such as MCN5 and CBI-2, with identified co-transmitters, revealed co-transmitter cooperativity necessary for motor pattern selection that would be missed in bath-application studies.

In some cases, neuropeptide and small molecule actions appear partially redundant. In the nematode *Caenorhabditis elegans*, serotonin or NLP-3 neuropeptide release from a modulatory PN is sufficient to activate egg-laying, however their combined actions elicit additional egg-laying. Further work is necessary to determine whether their actions converge onto the same targets (Brewer et al., 2019). Co-transmitters may converge onto the same cellular or even subcellular targets (Nadim and Bucher, 2014), however without cellular-level access to the full CPG circuit, similar network level actions may hide cellular divergence. In *Aplysia* feeding, three neuropeptides released from modulatory neuron CBI-12, each have the same circuit level effect, shortening the protraction phase of an ingestive motor pattern (Jing and Weiss, 2005; Zhang et al., 2018). However, the peptides act on different CPG neurons to mediate the same circuit effect (Zhang et al., 2018). Such redundancy may ensure a particular adjustment to circuit output even when some targets are unresponsive.

### 2.3. Spatial segregation of co-transmitter actions

Divergent co-transmitter actions may result from spatial segregation. In the crustacean STNS, modulatory PNs (MPN, PS) each use their peptide transmitter on CPG neurons within the stomatogastric ganglion (STG), but their small molecule transmitters act at distinct arbors, in different ganglia [commissural ganglia (CoGs)] (Figure 1C; Nusbaum and Marder, 1989b; Blitz and Nusbaum, 1999; Kwiatkowski et al., 2013). Spatially distinct actions could occur due to distinct trafficking of transmitter vesicles, differential receptor expression on postsynaptic targets, or differential sensitivity of transmitter release to neuronal activity (Kueh and Jellies, 2012; Nusbaum et al., 2017; Cropper et al., 2018; Cifuentes and Morales, 2021). Where determined, the low end of physiological firing frequencies is sufficient to release both peptide and small molecule transmitters (Cropper et al., 2018). On a finer scale, peptidases can constrain the actions of neuronally-released peptides, enabling distinct effects even when released into the same densely overlapping neuropil regions (Christie et al., 1997; Blitz et al., 1999; Nusbaum, 2002; Wood and Nusbaum, 2002; Nässel, 2009). Although neuromodulators are often considered to act *via* relatively non-specific “volume transmission,” it is becoming increasingly clear that there is also spatial constraint of neuromodulator actions (Disney and Higley, 2020; Liu et al., 2021; Nässel and Zandawala, 2022). Localization of reuptake and degradative machinery, and constrained release/receptor distributions beyond anatomically-defined synapses can limit the sphere of neuromodulator influence (Nusbaum, 2002; Disney and Higley, 2020; Liu et al., 2021; Eiden et al., 2022).

### 2.4. Local presynaptic feedback onto modulatory projection neurons

The ability to record from modulatory PN axon terminals revealed local presynaptic regulation of their transmission (Nusbaum, 1994). For example, rhythmic presynaptic inhibition from a circuit neuron onto modulatory PN terminals in the crab STNS and the subsequent waxing and waning of modulatory effects is essential to elicit a chewing pattern (Coleman et al., 1995). Further, the system is tuned such that this local feedback inhibition results in a more coordinated motor pattern when both PN copies are coactive compared to the same cumulative activity in a single PN copy (Colton et al., 2020). The presynaptic regulation occurs at terminals that are ∼1–2 cm distant from the soma (Figure 1A) and due to electrotonic decay, is not present in somatic recordings and does not alter PN activity initiating in the PN ganglion of origin (Nusbaum et al., 1992; Coleman and Nusbaum, 1994; Coleman et al., 1995). Local synaptic input includes chemical transmission between circuit neurons and PNs and between PNs, plus extensive electrical coupling between circuit neurons and PN terminals (Perrins and Weiss, 1998; Hurwitz et al., 2005; Stein et al., 2007; Marder et al., 2017; Blitz et al., 2019). Local feedback actions may generally alter transmission, or be more specific, including decreasing chemical but not electrical transmission (Coleman et al., 1995), or decreasing peptide but not small molecule transmitter release (DeLong et al., 2009). Rhythmic presynaptic regulation from CPG elements can also cause modulatory PN actions to occur *via* distinct mechanisms (e.g., electrical vs. chemical transmission) during different phases of motor output (Coleman et al., 1995; Hurwitz et al., 2005). Long-distance synaptic feedback also regulates PN transmission, however through changes in PN activity (see Section “3.3. Long-distance CPG feedback”). While much continues to be learned from bath-application studies, studies discussed above provide a note of caution, as even co-transmitter bath application may not mimic neuronal release due to the lack of spatial and temporal control that occurs with neuronally-released neuromodulators.

## 3. Regulation of modulatory projection neuron activity

Modulatory PNs serve as a link between sensory and/or higher-order inputs, and the motor circuits responsible for behavior. Thus, understanding how PN activity is controlled is important to understanding how sensory information and higher-order decisions are converted to appropriate behavioral responses.

### 3.1. State-dependence

**FIGURE 2:**
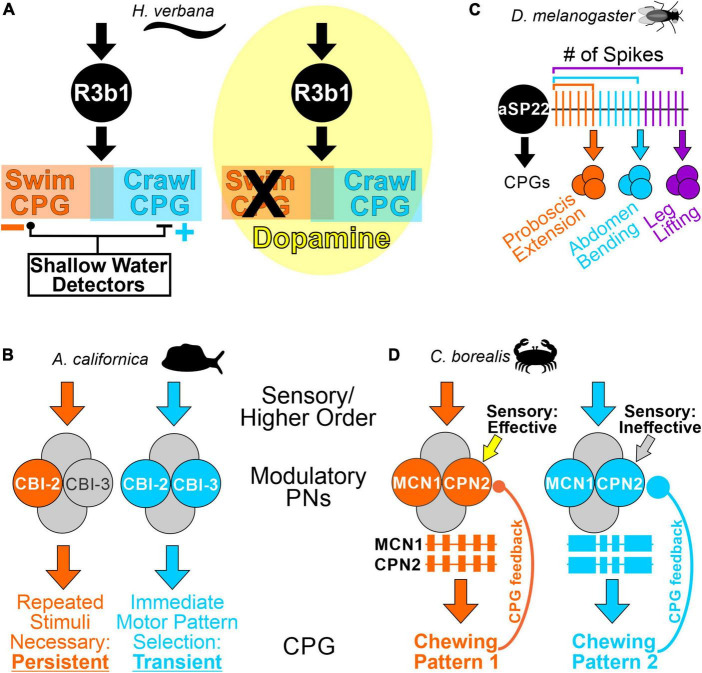
Motor pattern selection by modulatory PNs is state-dependent, and can be encoded in the population of active PNs, or in PN activity. **(A)** The effects of PN R3b1 are determined by environmental and internal conditions. *Left*, in an *in vitro* or semi-intact leech preparation, the R3b1 neuron elicits either swimming or crawling in response to the same input. The swim and crawl CPGs consist of partially overlapping neurons (orange and blue boxes). Fluid depth around the animal determines which locomotor pattern is selected. The proposed mechanism is that “shallow water detector” neurons provide inhibitory input to the swim CPG and excitatory input to the crawl CPG (Esch et al., 2002). *Right*, in the presence of dopamine (yellow cloud), the entire nervous system is biased toward crawling, and R3b1 only elicits crawling (Puhl et al., 2012). **(B)** Distinct subpopulations of activated PNs select feeding patterns with different dynamics. When the modulatory PN CBI-2 alone is activated, repeated stimulation is necessary to elicit an ingestive feeding pattern which persists for ∼30 min. However, if CBI-2 and CBI-3 are co-activated, an ingestive feeding pattern is immediately selected, but it is a transient activation (Evans et al., 2021). **(C)** The same PN, aSP22, activates different CPGs and different behaviors based on a spike number code. In this “ramp-to-threshold” example, as an increasing number of action potentials crosses different thresholds, aSP22 progressively activates CPGs contributing to different aspects of courtship (McKellar et al., 2019). **(D)** In response to different stimuli, the modulatory PNs MCN1 and CPN2 elicit qualitatively different chewing patterns due to distinctions in their activity patterns and rates (Beenhakker and Nusbaum, 2004; Blitz et al., 2008; White and Nusbaum, 2011; Diehl et al., 2013). MCN1 and CPN2 activity is indicated as extracellular recordings, with each colored box representing a burst of action potentials (different firing rates are not represented in the schematics). The differences in their activity are due to different strengths of CPG feedback (CPG feedback terminal size (colored circles) is representative of relative CPG feedback strength) (Blitz, 2017). Additionally, proprioceptive sensory neurons regulate MCN1 and CPN2 activity in the “orange” state when CPG feedback is weak, but not in the “blue” state, when CPG feedback is stronger (Beenhakker et al., 2007; White et al., 2017). Species used in the referenced studies are indicated in the panels.

*In vitro* and *in vivo*, single modality sensory input can be sufficient to initiate relevant behaviors *via* activation of identified modulatory PNs (Willard, 1981; Rosen et al., 1991; Horn et al., 1999; Jing and Weiss, 2005; Hedrich et al., 2011). However, PN activity is often regulated by multiple sources. In particular, inputs relaying behavioral state information can alter PN sensitivity to other inputs during ongoing behaviors, or result in different behavioral versions, on multiple time scales (Kristan and Shaw, 1997; Staudacher, 2001; Beenhakker et al., 2007; Barrière et al., 2008; White et al., 2017; Ache et al., 2019; Cook and Nusbaum, 2021). State-dependent PN activity may be a consequence of inputs specifically targeting PNs, such as courtship-promoting neurons converging with visual input onto the *Drosophila* P9 PN, to elicit courtship locomotor behavior (Bidaye et al., 2020). Behavioral state can also be conveyed to PNs through broadly-acting hormones (Willard, 1981; Mesce and Pierce-Shimomura, 2010; Flood et al., 2013). In the medicinal leech, circulating serotonin increases with hunger, coincident with a decreased threshold for swimming. Although serotonin does not activate swim-activating cell 204, it modulates its intrinsic properties, making it easier for other inputs to activate this neuron and elicit swimming (Angstadt and Friesen, 1993; Kristan et al., 2005). Even if the responsiveness of a modulatory PN does not change, the consequences of its activity may be state-dependent. The leech R3b1 PN elicits crawling or swimming, with the decision determined by the surrounding fluid level (Esch and Kristan, 2002). “Shallow water detector” sensory neurons appear to select motor output downstream from modulatory PNs, *via* actions on CPG neurons (Figure 2A). However, dopamine application biases the entire nervous system toward crawling and R3b1 only elicits crawling in this context (Figure 2A; Puhl et al., 2012), suggesting both PN- and CPG-level control of motor system state.

### 3.2. Long-lasting activity states

Inputs to modulatory PNs have rapid transient effects, *via* fast synaptic transmission, or trigger activity persisting beyond the stimulus duration, *via* slower metabotropic actions (Rosen et al., 1991; Beenhakker and Nusbaum, 2004; Kristan et al., 2005; Brodfuehrer et al., 2008; Benjamin, 2012). For long-lasting PN activation, a behavioral switch might require active termination of PN activity, such as a transient “stop” signal from a sensory pathway that triggers an incompatible behavior *via* other PNs (Esch and Kristan, 2002; Mesce and Pierce-Shimomura, 2010). Additionally, interactions between modulatory neurons, serving to either reinforce or suppress activity in other modulatory PNs, enables them to play important roles in maintaining or switching behavioral state. This includes inhibiting competing PNs to remove their drive of an alternative CPG, activating PNs which inhibit a competing CPG, or exciting complementary PNs (Blitz and Nusbaum, 1997, 1999; Crisp and Mesce, 2006; Wu et al., 2014; Pirger et al., 2021).

A persistent behavioral state can also occur without long-term PN activation, but instead due to the duration of PN modulatory actions. In *Aplysia* feeding, repeated CBI-2 stimulation progressively adapts CPG activity and improves behavioral output, due to second messenger accumulation in target CPG neurons (Cropper et al., 2017). As a result, the CPG is biased toward one output over another, which may stabilize the circuit when one behavior is more likely to be useful (Cropper et al., 2017). Different from this auto-regulation, in another mollusk, *Lymnaea*, the octopaminergic OC cells enhance CPG responses to other modulatory neurons for multiple motor pattern cycles (Benjamin, 2012). Thus, motor system state can be regulated directly at the PN level, or in circuit responsiveness to PNs, across multiple timescales.

### 3.3. Long-distance CPG feedback

Another source of regulation is synaptic feedback from CPG neurons to PNs, which results in PN firing being time-locked to circuit activity, including *in vivo* and in semi-intact preparations when PNs are activated by physiological stimuli (Gillette et al., 1978; Blitz and Nusbaum, 2008; Mesce et al., 2008; Hedrich et al., 2011; Blitz, 2017). A distinct case occurs in the stick insect *Carausius morosus* in which PN walking-timed activity is due to sensory feedback instead of CPG feedback (Stolz et al., 2019). Feedback to PNs contributes to inter-circuit coordination, duration of PN activity, and gating of other PN inputs (Wood et al., 2004; Antri et al., 2009; Kozlov et al., 2014). Additionally, feedback control of modulatory PN activity can be important for motor pattern selection (see Section “4.2. Activity code”).

## 4. Motor pattern selection

### 4.1. Population code

Although experimentally-induced activation of an individual PN can elicit a motor pattern, physiological stimuli often activate more than one PN type (Coleman and Nusbaum, 1994; Esch and Kristan, 2002; Beenhakker and Nusbaum, 2004; Benjamin, 2012; Follmann et al., 2018; Fahoum and Blitz, 2021). This raises the possibility that the “modulatory code” for selecting a motor output is one in which different stimuli activate distinct PN subsets, resulting in a combinatorial “population code.” Such a scenario occurs in several systems, and experimentally manipulating which PNs are active elicits switches between motor patterns (Kristan and Shaw, 1997; Combes et al., 1999; Kupfermann and Weiss, 2001; Hedrich et al., 2009; Guo et al., 2022). In *Aplysia* when the modulatory PN CBI-2 is active, repeated stimulations are necessary to elicit an ingestive pattern, which is persistent, but if CBI-2 and CBI-3 are both active, they immediately elicit an ingestive motor pattern without induction of a persistent state (Evans et al., 2021; Figure 2B). Thus, the population of modulatory neurons active can determine the pattern produced, and other aspects such as the dynamics of motor pattern selection.

### 4.2. Activity code

Quantitatively, modulatory PN firing rate can regulate motor output, although differences occur in network sensitivity (Kristan et al., 2005; Hedrich et al., 2011; Benjamin, 2012; Spencer and Blitz, 2016; Sakurai and Katz, 2019). Additionally, an “activity code,” i.e., PN pattern and/or rate can encode qualitatively distinct motor patterns and behaviors. In *Drosophila* courtship, the same descending PN (aSP22) uses cumulative spike count, to elicit different behaviors in a sequential fashion. In this “ramp-to-threshold” mechanism, different behavioral components of courtship are generated as the aSP22 spike count crosses a series of thresholds (Figure 2C; McKellar et al., 2019). In the crab STNS, mechanosensory neurons and neuroendocrine cells each trigger long-lasting activation of two modulatory PNs (MCN1, CPN2) (Beenhakker and Nusbaum, 2004; Blitz et al., 2008). However, differential, long-lasting, modulation of CPG feedback in these two states results in distinct MCN1/CPN2 activity patterns and rates which encode different chewing behaviors, and different sensitivity to sensory feedback (Figure 2D; Beenhakker et al., 2007; Blitz and Nusbaum, 2008, 2012; Diehl et al., 2013; Blitz, 2017; White et al., 2017). The ability to manipulate feedback synapses onto small populations of identified modulatory neurons was essential for these insights into how CPG feedback to PNs contributes to motor pattern selection. Collectively, these examples illustrate that the same PNs can use an activity code to select motor outputs, instead of a population code of different PN subsets, with both mechanisms possible even in the same system, albeit in distinct species (Beenhakker and Nusbaum, 2004; Blitz et al., 2008; Hedrich et al., 2009).

## 5. Conclusion

Cellular-level access to modulatory PNs at their somata and axon terminals, and their CPG neuron targets in several invertebrate preparations enabled insights into regulation of PN activity, strategies for selecting an appropriate motor pattern, and significant complexity in communication between modulatory PNs and their CPG targets. Invertebrate PNs and larger vertebrate populations similarly link sensory and higher-order processing with motor circuits, and many of the insights discussed have already, or likely will be found to extend to larger circuits (Dickinson, 2006; Sharples et al., 2014; Yang et al., 2020). Technological advances are enabling recording and manipulation of genetically identified populations in organisms with barriers to electrophysiological approaches (e.g., neuronal size, accessibility, population size). However invertebrate organisms remain important for determining how modulatory PNs regulate circuits at the cellular-level, *via* electrophysiological recordings and manipulations that remain difficult in larger systems. Given the rapidly developing techniques making investigation in larger systems more tractable, plus the application of genetic approaches to classic neurophysiologically-accessible model organisms (Kim et al., 2017; Northcutt et al., 2018, 2019; Devineni and Scaplen, 2022; Leiras et al., 2022), diverse models and approaches are expected to continue increasing our understanding of how motor circuits rapidly adapt to the everchanging conditions in and around us.

## Author contributions

DB wrote the first draft of the manuscript, revised the manuscript, read, and approved the final version.
